# Diffusion, search and attack motions of antibodies

**DOI:** 10.1038/s42003-025-08995-9

**Published:** 2025-10-13

**Authors:** Ralf Biehl, Margarita Kruteva, Orsolya Czakkel, Ingo Hoffmann, Dieter Richter, Andreas M. Stadler

**Affiliations:** 1 Jülich Centre for Neutron Science, Forschungszentrum Jülich GmbH, Leo-Brandt Strasse, 52428 Jülich, Germany; 2 Institut Laue-Langevin, 71 Avenue des Martyrs, 38042 Grenoble, France; 3 Institute of Physical Chemistry, RWTH Aachen University, Landoltweg 2, Aachen, Germany; 4 LINXS Institute of advanced Neutron and X-ray Science, Mesongatan 4, 224 84 Lund, Sweden

**Keywords:** Deformation dynamics, SAXS, Molecular conformation

## Abstract

A fundamental feature of the antibody structure is the flexible linker between the 3 fragments that allows great flexibility and simultaneous binding to epitopes of antigens and receptors. Combining dynamic light scattering, neutron spin-echo spectroscopy and PFG-NMR we determine characteristic internal fragment dynamics on top of translational and rotational diffusion under crowding conditions. Short-time and long-time translational diffusion show an effective hard sphere like behavior within a colloidal picture. Internal fragment motions are characterized as “attack” and “search” motions complemented by rotational fragment motions. We find that the “attack” motions exposing the binding domain are highly preserved from low to physiologically relevant concentrations and higher, while “search” motions and overall rotational diffusion are suppressed under crowding conditions. Hydrodynamic interactions change the friction between fragments determining relaxation times while interparticle interactions influence the strength of the entropic spring between fragments. The strategic redesign of the linker region to facilitate “attack” motions and fragment rotation has the potential to enhance the therapeutic efficacy of mAbs.

**Figure:**
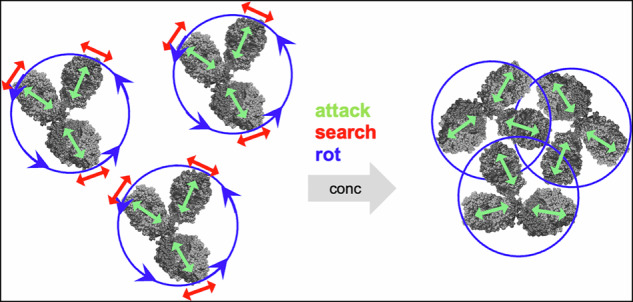

## Abstract (web summary)

The flexible linker in antibodies enables internal fragment dynamics. Under crowding conditions, we find that “attack” motions that expose the binding domain are preserved while “search” motions and rotational diffusion are suppressed.

## Introduction

**Fig. 1:**
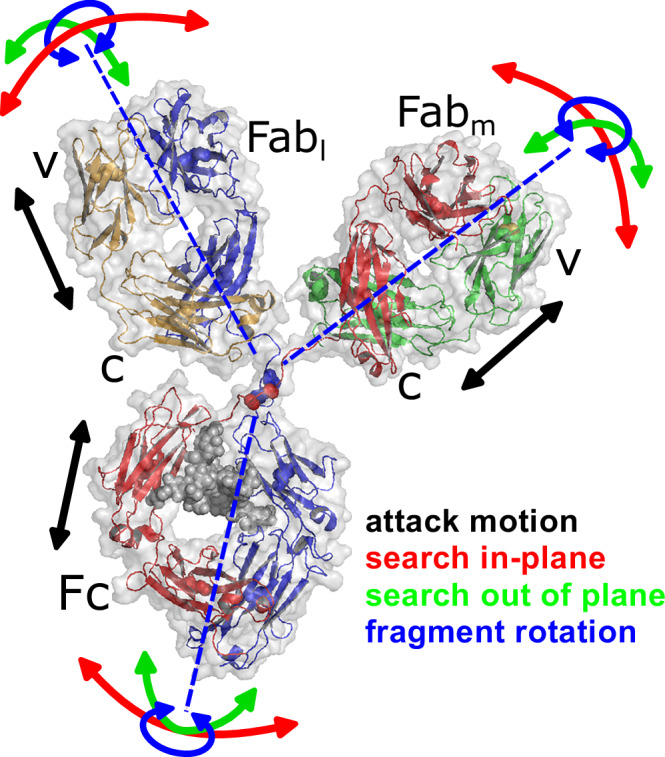
NISTmAb Primary Sample 8670^52^ in a SAXS refined configuration as described in the text (van der Waals surface in gray). Parts of the heavy chains (red, blue) together with bound glycans (gray spheres) build the Fc fragment while light chains (green, orange) with the other part of the heavy chains build the Fab fragments. Fc and Fab fragments, each with a molecular weight of about 50 kDa, are connected by the linker region built from a disordered region of the heavy chains (residues 222–239) where the amino acid motif CPPC (cysteine–proline–proline–cysteine, residues 229–232) stabilizes the linker with 2 disulfide bonds between cysteines (spheres in linker region). Fab fragments show a variable (V) and constant (C) region. The bounding sphere of mAb has a radius of 8.8 nm and of fragments ≈4.2 nm. Arrows indicate individual degrees of freedom for movements as “attack” motions (black), in plane bending (red), out of plane bending (green) and rotation around a fragment axis (blue). The figures were created using PyMOL (www.pymol.org).

Antibodies, also known as immunoglobulins (Ig), are the major component of our immune system against bacteria, viruses or toxins responsible for recognition of antigens and initialization of immune response. While different isotypes like the dimeric IgA or pentameric IgM exist, they share a common Y-shape structure (see Fig. 1) found in the most abundant isotype immunoglobulin G (IgG)^1,2^. The crystallizable fragment (Fc) as the trunk, can modulate immune response and communicate with effector cells via Fc receptors. Two antigen binding fragments (Fab_l_, Fab_m_) form the arms with the variable fragments at the top containing the antigen binding site, the paratope. The fragments are connected by a flexible linker region that allows configurational freedom and an extreme flexibility e.g., allowing the Fab to bind to epitopes separated by ~18 nm^3^.The variable region (V) is adapted to a specific antigen and is responsible for the specificity of the Ig with high binding affinity. This specificity makes monoclonal antibodies (mAb) extremely useful for various applications in biosensors, immunoassays (COVID, insulin) or as therapeutic antibodies^2,4^.

Over the years, an extensive amount of research has been done to understand the structure and flexibility of antibodies^5–11^, including the role of the hinge region^12^. It is anticipated that the flexibility of the hinge region modulates activity^13,14^ while hinge deletion obliterates binding to Fc gamma receptors but leaving the ability to bind to the antigen^15^_._ Hinge engineering has been proposed to optimize antibody activity^16^.

Administration of mAb as therapeutics is usually done by injection^17^. A major pharmaceutical challenge is the formulation of mAb solutions to improve bioavailability, enhance formulation stability, preserve biological function, and achieve a formulation viscosity suitable for injection. Main characteristics for administration of a larger amount of mAb within a relatively small volume (≈ 1–5 ml) are the isoelectric point, concentration and excipients in the formulation to enhance bioavailability without altering activity. Typical concentrations for administration are below 30 mg/ml, but higher concentrations up to 150 mg/ml are strongly desirable in pharmaceutical products, which challenge aspects like solubility, viscosity, phase separation or multimerization^18^. While the above drug formulations are more related to self-crowding, Ig is active in the blood plasma, interacting with all components present in plasma, changing the crowding conditions. Blood plasma contains proteins e.g., serum albumins, globulins or fibrinogens at a high concentration of 60–80 mg/ml together with electrolytes, hormones, carbon dioxide and oxygen. Ig contributes 20% to the protein fraction in plasma (named globulins). Crowding alters diffusion, solubility, phase separation, and self-association, as well as binding equilibria and reaction rates^19–24^. Viscosity is influenced by clustering where colloidal approaches are used to describe the viscosity increase at larger concentrations^25–27^.

The conformational dynamics of a multidomain protein like Ig is difficult to access. Methods like Förster resonance energy transfer (FRET)^28^ or double electron-electron resonance spectroscopy (DEER)^29^ need specific labeling to assess the dynamics between fragments. NMR based methods require isotopic labeling and focus more on local dynamics of fragments and are challenging for larger proteins^30–32^. Fluorescence anisotropy can be used to extract the rotational diffusion and separate different relaxation times related to fragment tumbling^33,34^. Other methods, such as small-angle X-ray or neutron scattering (SAXS/SANS) observe average ensemble structures in the solution, while electron microscopy or crystallography are limited to frozen configurations. Quasielastic neutron scattering methods like backscattering and time of flight methods can examine atomic motions on nanosecond to picosecond timescale^35–38^. Neutron spin-echo spectroscopy (NSE) is a label-free high-resolution inelastic scattering technique that covers the most interesting time scales from 0.01 to 500 ns and length scales from 30 to 2 nm. The ability to concurrently resolve time and space is the key to identify distinctive spatial patterns of specific domain motions that superpose translational and rotational diffusion for structured and intrinsically unfolded proteins^39–42^.

In the present work, we combine methods working on different time and length scales, such as dynamic light scattering (DLS), pulsed field gradient NMR (PFG-NMR) and NSE, to obtain a comprehensive picture of mAb fragment dynamics at concentrations relevant for their function and for the application of antibodies as drugs. The different time and length scales make it necessary to relate these by a theoretical framework in terms of a colloidal theory using a spherical approximation that includes direct and hydrodynamic interactions (HI). Colloidal theory was already successfully used for globular proteins and mAb^39,43–46^, but shows also limitations when proteins are described as effective spheres at larger concentrations^47,48^.

Colloidal theory ranges from short-time dynamics, where the center of mass diffusion does not significantly change positions, to long times, where the entire neighborhood changes and explains the transition from collective diffusion on large length scales to self-diffusion on shorter length scales. Our results prove the surprising quality of the spherical approximation describing the translational center of mass diffusion if a hydrodynamic interaction radius R_HI_ is introduced. The real shape seems to be unimportant on large length scales and for long times. The inclusion of direct interactions by appropriate structure factors is central to colloidal theory.

On mid-range length scales of fragment distances, NSE is sensitive to rotational diffusion and fragment dynamics. Colloidal theory is complemented by modeling internal fragment motions by coherent mode form factors that describe the internal degrees of freedom of rigid fragments connected by a disordered linker, similar to elastic normal mode analysis and principal component analysis (PCA)^49–51^. These fragment modes are assumed to be decoupled from each other and overall diffusion. The model distinguishes different motional patterns that can be recognized in the coherent intermediate scattering function as measured by NSE. The evolving detailed picture of internal fragment motions is characterized descriptively as forward “attack” motion and lateral “search” motion. For higher concentrations, the “search” motions are severely restricted while “attack” motions are kept functional at all concentrations. The detailed modeling of internal motions allows to access the short-time self-diffusion of the anisotropically flexible mAb that is difficult to measure by other methods.

In the following, we first examine the static structure of the used mAb by SAXS to yield a form factor and structure factor that allows us to model a reasonable equilibrium mAb configuration. Collective and self-diffusion is afterwards examined by DLS and PFG-NMR within the observed concentration range to get a comprehensive picture of the differences. The following section describes the NSE measurements of short-time dynamics, which, in conjunction with DLS, allow access to short-time diffusion and fragment dynamics. Finally, the link to long-time diffusion is described. Our conclusions discuss the implications of fragment dynamics for the biological function of the mAb.

## Results

### mAb structure analysis and interactions

**Fig. 2:**
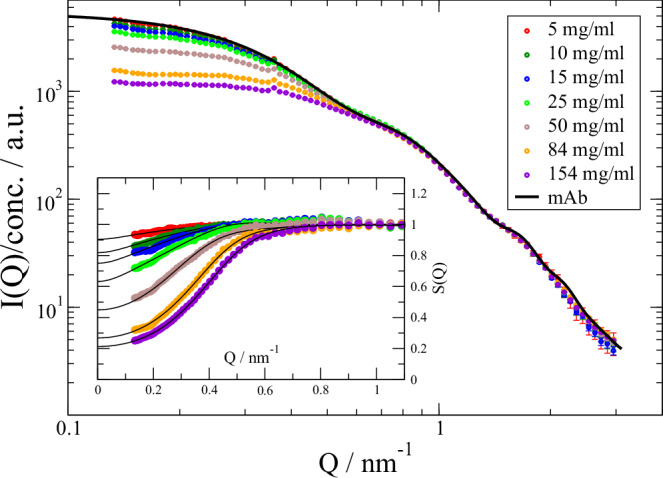
SAXS analysis of mAb presenting concentration scaled scattering intensities after background subtraction for *T* = 25 °C. The form factor *P*_mAb_(*Q*) was extracted by extrapolation of concentrations ≤25 mg/ml to *conc* = 0. The form factor is fitted within a differential evolution algorithm and refined by a Levenberg-Marquardt fit (black line). The experimental form factor at higher *Q* is independent of concentration, which demonstrates that the average configuration of mAb is independent of the distance to the neighboring mAb. The inset shows the revealed SFs using $I(Q) \sim c{S}^{{\prime} }\left(Q\right){P}_{{mAb}}(Q)$. Lines show fits using a RMSA SF^54^ for conc. ≤25 mg/ml and a two-Yukawa potential^55^ for *conc*.>25 mg/ml allowing for individual potential strength of the attractive and repulsive components. See SI for details about the RMSA and two-Yukawa potential and resulting SF parameters. Error bars represent standard deviation of the mean.

As a prototype for Ig, we use the NISTmAb monoclonal antibody (mAb)^52,53^ as provided by National Institute of Standards and Technology (NIST) within the “LINXS Antibodies in Solution Research Program”. Figure 2 presents concentration-scaled SAXS data over a concentration range from 5 to 155 mg/ml in an aqueous acetate buffer at pH 5. The scattered intensity *I*$\left(Q\right) \sim P\left(Q\right){S}^{{\prime} }(Q)$ depends on the form factor *P(Q)* describing the configuration and apparent structure factor ${S}^{{\prime} }(Q)$ describing interactions between mAb (see Methods). For lower concentrations ≤25 mg/ml *S*(*Q*) can be well described by the RMSA^54^ SF describing a charged particle in solution with a screening electrolyte. For larger concentrations the incorporation of attractive interactions within a two-Yukawa potential^55^ and an increase in concentration by a factor 3 was needed. The increase corresponds to the change of protein number density to fragment number density reflecting a crossover from protein interaction to fragment interactions. The crossover between these regimes coincides also with the concentration of 33 mg/ml when the mAb bounding spheres with a radius of ≈8.8 nm just touches on average. The 3-fragment character with nearly equal fragment size becomes more important and attractive contributions from hydrophobic patches at the surfaces get significant.

The NIST mAb PDB file is an artificial structure using experimental structures for Fc and Fab fragments that are randomly positioned and connected by a randomly positioned linker region^52^. The disordered linker allows the three fragments to dynamically fluctuate in their configurational space. Assuming a single equilibrium configuration, we can use characteristic fragment displacements to find a reasonable equilibrium configuration and to describe fragment dynamics as relaxation along these displacements. This concept is successfully used in MD simulation as PCA or based on a given protein structure in normal mode analysis^49,50^. Normal mode analysis would result in mode displacements that strongly depend on the initial, here artificial, configuration of the linker. In a simplified approach, we assume three translational and one rotational internal degree of freedom (iDOF) for each fragment within the reference frame of the whole mAb and additional linker scaling and bending of the Fc fragment. These characteristic displacements are first used to find a suitable equilibrium configuration that fits the mAb form factor *P*(*Q*), and the iDOF are later used to describe the fragment dynamics.

Based on the NIST PDB structure we allow the linker region to shrink and let the fragments bend independently within the plane of the three fragments and perpendicular to it around the central CPPC motif of the linker (see Fig. 1) describing two translational iDOF (see Fig. 1 red and green arrows). These “search” motions change the relative orientation between fragments. We alter the distance of individual fragments from the central CPPC motif as a third translational iDOF (see Fig. 1 black arrows). These “attack” motions expose the binding region at the end of the Fab. The rotational iDOF is an axial rotation of a fragment around the connection line of the CPPC center and its center (see Fig. 1 blue arrows). To fit the equilibrium configuration to a SAXS form factor the Fc fragment is additionally bent around the connecting line between the two Glu236 of the heavy chains where the linker is connected to the Fc fragment and, at last, the linker is scaled and positioned to reasonably reconstruct the linker. The details of the disordered linker have only a minor contribution to the scattering with 38 compared to 1326 amino acid in total.

The result is shown in Fig. 2 as line and the corresponding refined structure is presented in Fig. 1. The structure is more compact with a radius of gyration *R*_g_ = 5.03 ± 0.05 nm compared to the NIST mAb structure with 5.6 nm. Any ensemble description of a corresponding form factor cannot contain additional information about configurational flexibility if a single structure can describe the measurement. On the other hand, it is assumed that the equilibrium structure is an average within the configurational ensemble. The previous analysis assumes that the form factor is independent on concentration. This assumption cannot be made in general but for mAb smaller configurational changes would be visible at *Q* > 0.7 nm^-1^ where *S*(*Q*) = 1. As these are not observed our assumption is appropriate.

In the following we will demonstrate that direct measurements of fragment movements by NSE allow us to discriminate different types of motion around the equilibrium structure, their amplitudes and timescale.

### Long-time collective diffusion (DLS) and long-time self-diffusion (PFG-NMR)

**Fig. 3:**
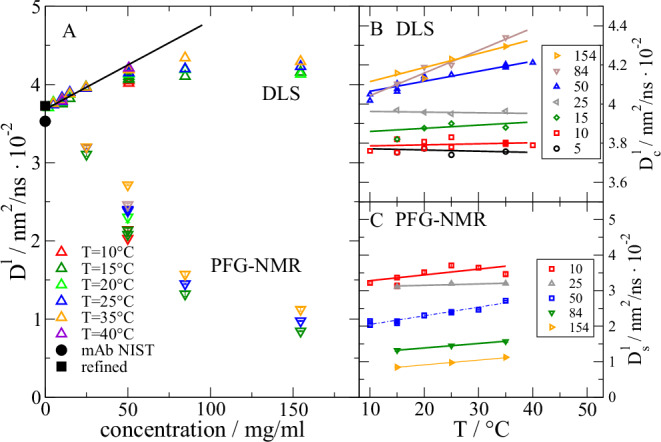
A: Rescaled collective diffusion ${D}_{c}^{l}$ (up triangles) measured by DLS and self-diffusion ${D}_{s}^{l}$ (down triangle) measured by PFG-NMR for various concentrations and temperatures as indicated. Measured values are all scaled by *kT*/*η* with viscosity *η* to the reference temperature *T* = 25° C. Additionally, the calculated translational diffusion coefficient based on the original NIST PDB structure and based on the SAXS refined PDB structure are shown (solid black circle and square). Extrapolation to ${D}_{0}$ for c < 25 mg/ml is indicated as black line. B: Temperature dependence of DLS measured ${D}_{c}^{l}$ for all concentrations given in mg/ml in the legend. C: Temperature dependence of PFG-NMR measured ${D}_{s}^{l}$. Same symbols and concentrations as in B. Errors indicate 1-sigma error from the fit (see Materials and Methods).

To examine the dynamics of mAb we first look at the collective diffusion measured by DLS and the long-time self-diffusion measured by PFG-NMR presented in Fig. 3 for temperatures in the range 10-40 °C. PFG-NMR measures the long-time self-diffusion ${D}_{s}^{l}$ (see Methods and SI). DLS measures the intensity correlation function with relaxation times typically in the range of some 10 microseconds for mAb at *Q* = 0.026 nm^-1^ resulting in the long-time collective translational diffusion ${D}_{c}^{l}$ for times ${{{\rm{\tau }}}}\, \gg \, {\tau }_{l}=\frac{{R}_{h}^{2}}{{D}_{0}}\approx 700$ ${{{\rm{ns}}}}$ where ${R}_{h}=5.4$ nm is the hydrodynamic radius and ${D}_{0}$ the single protein translational diffusion coefficient assuming validity of the Stokes-Einstein relation^44^. For the concentrations considered in this work (5–154 mg/ml) the difference between short and long-time collective diffusion is small (<3%) and for small non-pairwise additive HI practically ${D}_{c}^{l}\cong {D}_{c}^{s}={D}_{c}$ ^44,56,57^.

${D}_{0}$ is determined according to ${D}_{c}^{l}(c)={D}_{0}(1+{k}_{D}c)$ with the interaction parameter ${k}_{D}$ for low concentrations (see Fig. 3A). The resulting ${D}_{0}$ = 0.0368 nm^2^/ns fits to the expected diffusion coefficient of the refined structure 0.0368 nm^2^/ns, which is slightly larger than for the NIST PDB structure (0.0353 nm^2^/ns) both calculated using HYDROPRO^58,59^. The larger value indicates here a more compact structure corroborating the SAXS finding. The increase of ${D}_{c}$ with concentration results from a dominant repulsive interaction between the mAb that stabilizes the mAb solution. For concentrations >25 mg/ml the increase falls back behind the linear increase and stays on a more constant value. For ${D}_{s}^{l}$ we observe a general decrease. Both observations are expected and can be described within a colloidal picture^44^. To describe the difference between collective and self-diffusion we use methods related to short-time dynamics based on direct interactions subsumed in *S*(*Q*) and HI leading in the short-time limit to the correction ${D}_{c}^{s}={D}_{0}H(Q)/S\left(Q\right)$ as explained later. For DLS and PFG-NMR we find similar temperature trends. After correction of viscosity effects, at lower concentrations the diffusion is constant. At concentrations >25 mg/ml we find an increase with increased temperature. The observed differences are reproducible and reversible as proved by repeated measurements cycling the temperature for 50 mg/ml. As the SAXS form factor of the mAb is not changed by temperature (see SI Fig. S1) it is reasonable that the interparticle interactions and/or HI change with temperature, which is only significant at larger concentrations when fragments motions become noteworthy.

### Short-time collective diffusion (NSE)

**Fig. 4:**
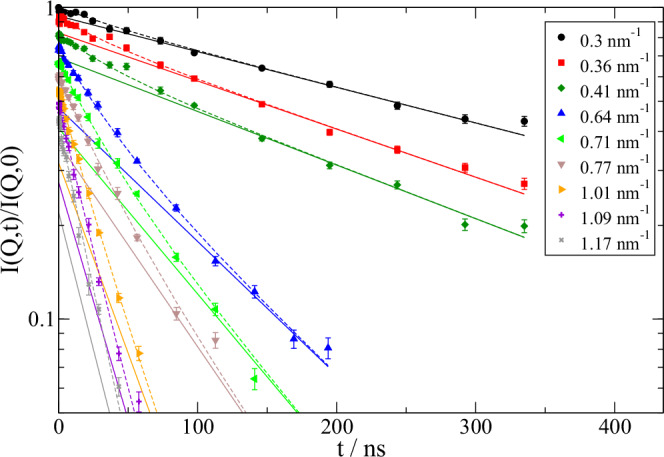
NSE data for 84 mg/ml with corresponding fit showing a fast relaxation on top of a slow relaxation for selected *Q.* Data are shifted consecutively for clarity. Solid lines describe the long-time diffusion ${D}_{{slow}}$ extrapolated to short times. Broken lines show the full fit result. Fit parameters are given in Table 1 and Fig. 5. Error bars represent standard deviation of the mean.

The coherent normalized intermediate dynamic structure factor *I*(*Q*,*t*)/*I*(*Q*,0) measured by NSE in the short-time limit is determined by translational and rotational diffusion and internal dynamics like fragment motions. Figure 4 shows exemplary spectra for a concentration of 100 mg/ml of mAb (see SI for other concentrations). Obviously, *I*(*Q*,*t*)/*I*(*Q*,0) is not a single exponential like $\sim \exp (-{Q}^{2}D(Q)t)$.

### Fast “attack” motion contribution

We assume specific fast modes of internal motions on top of overall slower diffusion and additional slow fragment dynamics described by *D*_slow_(*Q*) using [30,33,41].

1 $$\frac{I\left(Q,t\right)}{I(Q,o)}=\left(\left(1-A(Q)\right)+A\left(Q\right){e}^{-t/\lambda }\right){e}^{-{Q}^{2}{D}_{{slow}}\left(Q\right)t}$$

Here *Q* is the scattering vector, *A*(*Q*) is the amplitude of included fast fragment motions in a small displacement approximation for normal modes (see Methods). We assume an overdamped relaxation of the modes with a common relaxation time *λ*.

As shown previously, the linker region acts as an entropic spring^40^. While “search” motions (bending) do not alter the linker length, “attack” motions significantly alter the linker length, thereby inducing a restoring force. Consequently, “attack” motions are expected to be faster and “search” motions are expected to diffuse slowly in a shallow potential created by the other fragments. We sum the “attack” motions of the three fragments to determine *A*(*Q*) for fitting (see Methods), resulting in a common root mean square displacement ${u}_{f}$, a relaxation time *λ* and a common *Q*-dependent diffusion coefficient *D*_slow_(*Q*).

**Fig. 5:**
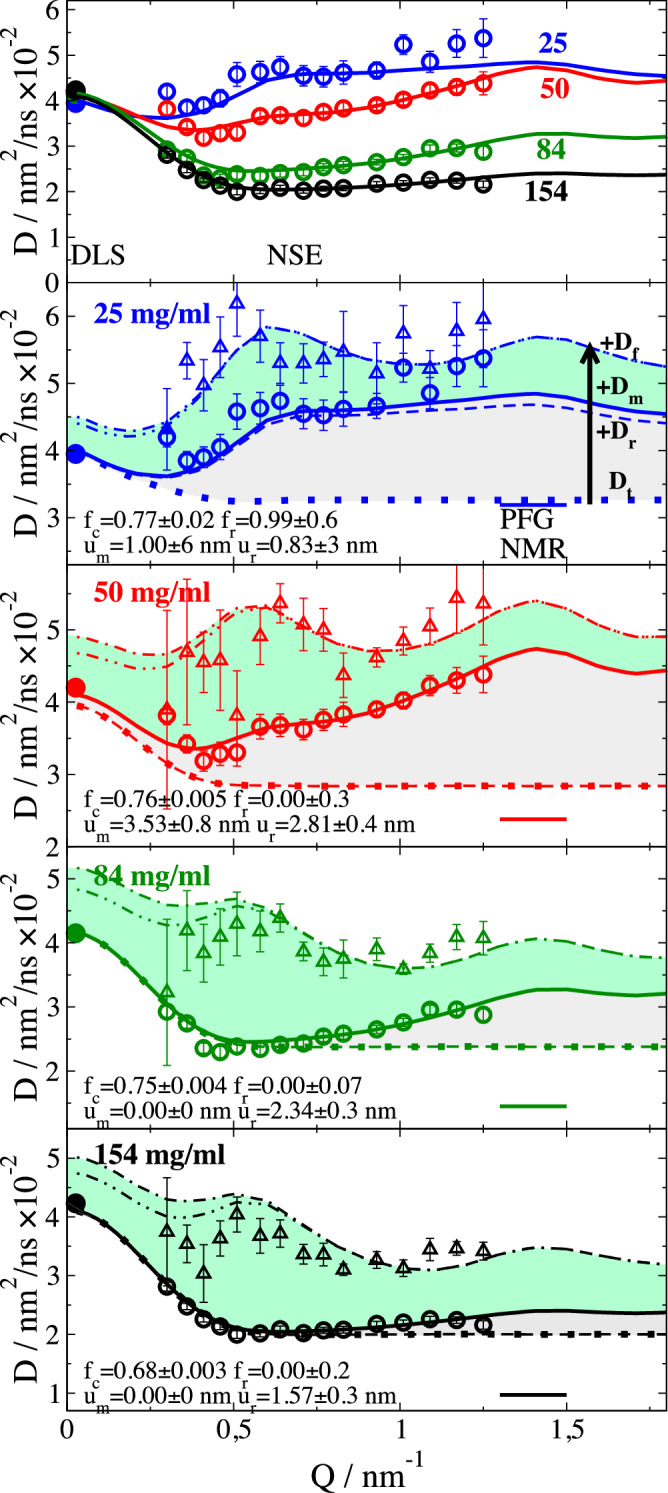
Long-time diffusion *D*_slow_(*Q*) for mAb with indicated concentrations (color coded like top panel). The top overview shows NSE measured *D*_slow_(*Q*) (open symbols) together with DLS measured ${D}_{c}^{l}$ (full symbols at Q = 0.026 nm^-1^) for direct comparison. The lower panels show for indicated concentrations *D*_slow_(*Q*) and ${D}_{c}^{l}$ together with *D*_cum_(*Q*) from a cumulant fit (open triangles) for t < 20 ns representing the short time limit of the NSE spectra in Fig. 4 and Figure S2. All panels show the modeled contribution from translational diffusion *D*_t(_*Q*) (dotted line), including rotational diffusion *D*_r_(*Q*) (dashed line) and including *D*_m_(*Q*) as mode contributions from “search” motions and fragment rotation (solid line). With the additional contribution from fast “attack” motions *D*_f_(*Q*) (dash-dotted line) a similar pattern as *D*_cum_(*Q*) is observe_d_. H_d_D_f_(*Q*) is used to include hydrodynamic interaction (dash-double-dotted line). Gray area highlights contributions from *D*_r_(*Q*), “search” motion and fragment rotation, while the light green area highlights the contribution from “attack” motions *D*_f_(*Q*). PFG-NMR measured ${D}_{s}^{l}$ is presented asa short line at *Q* ≈ 1.4 nm^-1^ for direct comparison to ${D}_{s}^{s}$ apparent at high *Q* in *D*_t_(*Q*). Fitted scaling coefficients f_c_, f_r_ and root mean square displacements from rotation u_r_ and displacements u_m_ are noted as written text in the respective figures. For vanishing rotational diffusion *D*_t_ and +D_r_ lines fall together for concentrations ≥50 mg/ml. Error represents 1-sigma errors from the fit.

Fit results are presented in Fig. 4 and Fig. S2 in SI. We find in general excellent fits that describe a fast “attack” motion on 25–55 ns relaxation time and ${u}_{f}$ between 0.6–1.2 nm. Fit parameters are given in Table 1 and ${D}_{{slow}}({{{\rm{Q}}}})$ is presented in Fig. 5.

**Table 1:** Fit parameters related to the fast relaxation for all concentrations

c mg/ml	${u}_{f}$ nm	$\lambda$ ns	k_f_ g/ps^2^/mol	ξ kg/ps/mol
25	0.64 ± 0.18	26 ± 14	2.46 ± 0.48	87 ± 13
50	1.16 ± 0.18	55 ± 12	0.75 ± 0.11	70 ± 6
84	1.2 ± 0.09	47 ± 5	0.65 ± 0.07	61 ± 5
154	0.89 ± 0.05	28 ± 3	1.1 ± 0.08	67 ± 4

Errors are 1-sigma errors. Total force k_f_ and total friction ξ refer to a fragment. Unit conversion to 1 pN/nm = 1.66 g/ps^2^/mol.

The initial fast relaxation from “attack” motions has a stronger amplitude at 50 and 84 mg/ml concentration with *rmsd* > 1 nm compared to lower and highest concentrations. The relaxation time increases at the same time from ≈26 ns to ≈50 ns.

To relate the observation to forces and friction, we assume Brownian motion of the fragments in a harmonic potential around the equilibrium positions, a problem described by the Ornstein-Uhlenbeck process^60,61^. The corresponding model for the coherent intermediate scattering function ${I}_{{OU}}\left(Q,t\right)$ (see Methods) allows us to fit the fast relaxation process and to determine the associated force constant and the friction exerted on the fragments. The resulting parameters are given in Table 1. We observe that with increasing concentration the friction is decreasing from 87 kg/ps/mol to about 65 kg/ps/mol. The friction of a free fragment with the solvent ${\xi }_{0}^{{frag}}$ can be deduced from the free diffusion as ${\xi }_{0}^{{frag}}={k}_{B}T/{D}_{0}^{{frag}}$ with ${D}_{0}^{{frag}}$ calculated by HYDROPRO^58,59^ and is on the order of 41 kg/ps/mol. The observed much stronger friction of the fragments might result from HI between the fragments. With increasing concentration, respectively, closer neighboring mAb, the HI seem to be reduced. *rmsd* are directly related to the width of the potential and show that a softer potential leads to larger fluctuations. We might compare k_f_ to an entropic spring built of the linker with ${k}_{f}^{{entropic}}={k}_{b}T/N/{b}^{2}$. The linker length to the CPPC motive is *N* ≈ 8 while the length to another fragment is *N* ≈ 18. With a monomer length *b* ≈ 0.38 nm this leads to *k*_f_ ≈ 2.8–6.4 g/ps^2^/mol. While *k*_f_ for the lowest concentration seems reasonable for a full-length entropic spring, *k*_f_ for higher concentrations is smaller and indicates an attractive potential of other mAb that partially compensate the entropic spring directed toward the center. The attractive component was already observed within the analysis of the SF and can be related to hydrophobic patches or opposite charges at opposing surfaces.

### Slow diffusion: translational and rotational diffusion

The slow diffusion component ${D}_{{slow}}(Q)$ is presented in Fig. 5 in comparison to DLS and PFG-NMR measured data at 25°C. The NSE measured ${D}_{{slow}}(Q)$ show a general decrease with increased concentration, like PFG-NMR, but different to the slowly increasing DLS data. For larger concentrations, a clear increase in lower *Q* is seen that extrapolates to the DLS data. We will now describe the additive contributions to the slow diffusion component

2 $${D}_{{slow}}(Q){=D}_{c}^{s}\left(Q\right)+{D}_{r}(Q){+D}_{{{{\rm{m}}}},{{{\rm{search}}}}}\left(Q\right)+{D}_{{{{\rm{m}}}},{{{\rm{frag\; rot}}}}}\left(Q\right)$$

from translational diffusion ${D}_{c}^{s}\left(Q\right)$, rotational diffusion *D*_r_(*Q*) and internal dynamics from “search” motions and fragment rotation *D*_m_(*Q*). We fit the different components contributing to ${D}_{{slow}}(Q)$ seen by DLS and NSE as presented in Fig. 5 using the corresponding fit parameters *f*_*c*_, *f*_*r*_, *u*_*m*_ and *u*_*r*_ as described in the following. Finally, we discuss the fast “attack” mode contribution *D*_f_(*Q*).

To describe the transition between ${D}_{c}$ of DLS and intermediate *Q* NSE data, we resort to analytical colloidal theory for spherical particles. For interparticle interactions like the correction for colloidal spherical particles e.g., we have ${D}_{c}^{s}\left(Q\right)={D}_{0}H\left(Q\right)/{{{\rm{S}}}}\left(Q\right)$. This implies the assumption that *S*(*Q*) and the hydrodynamic function *H*(*Q*) describe a kind of configurational ensemble average and the decoupling of center of mass diffusion and internal dynamics. An analytical method to calculate *H*(*Q*) is the δγ-expansion of Beenakker and Mazur for spherical particles of radius *R* (see Methods). *H*(*Q*) has a similar shape as *S*(*Q*) but with smaller amplitude and a high *Q* limiting value of ${D}_{s}^{s}/{D}_{0}$. We fit here the effective HI radius ${R}_{{HI}}={{f}_{c}R}_{h}$ as a fraction ${f}_{c}$ of the hydrodynamic radius *R*_h_ of diluted mAb, assuming that HI are averaged over mAb orientations and can be represented by an effective sphere radius^62^.

The resulting components of the collective translational diffusion *D*_c_(*Q*) are shown in Fig. 5 as pointed line. For smaller *Q* we see the increase towards low Q due to ${D}_{0}H\left(Q\right)/S\left(Q\right)$. For larger *Q* > 0.5 nm^-1^ *S*(*Q*) ≈ 1 and we observe the short-time self-diffusion ${D}_{s}^{s}$ as a constant value. The scaling factor *f*_c_ presents a small concentration dependence and is essentially constant, giving an effective hydrodynamic interaction radius of ${R}_{{HI}}\approx {3/4R}_{h}$.

With the 6×6 diffusion matrix ***D*** calculated by HYDROPRO^58,59^, the translational/rotational diffusion ${D}_{0}(Q)$ of a rigid protein can be calculated^39^:

3 $${D}_{0}\left(Q\right)=\frac{1}{{Q}^{2}P\left(Q\right)}\left\langle \rho \left({{{\boldsymbol{Q}}}}\right)\left(\begin{array}{c}{{{\boldsymbol{Q}}}}\\ {{{\boldsymbol{Q}}}}\times {{{{\boldsymbol{r}}}}}_{k}\end{array}\right){{{\boldsymbol{D}}}}\left(\begin{array}{c}{{{\boldsymbol{Q}}}}\\ {{{\boldsymbol{Q}}}}\times {{{{\boldsymbol{r}}}}}_{l}\end{array}\right){\rho }^{* }\left({{{\boldsymbol{Q}}}}\right)\right\rangle$$

While the constant translational part ${D}_{0}$ needs to be corrected by the above *H*(*Q*)/*S*(*Q*) correction, the rotational contribution ${{D}_{r}\left(Q\right)={D}_{r0}\left(Q\right)-D}_{0}$ is rescaled according to ${D}_{r}\left(Q\right)={f}_{r}{D}_{r0}(Q)({1-0.631\Phi }_{{{{\rm{HI}}}}}-0.726{\Phi }_{{HI}}^{2})$ ^63^ using the HI volume fraction ${\phi }_{{HI}}$ from the ${D}_{c}^{s}$ correction. The rotational correlation time is ${\tau }_{r0}=1/6{D}_{r0}=213$ ${ns}$. We fit the factor f_r_ that accounts for a possible reduction of the rotational diffusion compared to the colloidal case. For concentration of 25 mg/ml we find no reduction *f*_c_ ≈ 0.99 (dashed line) which contributes a large fraction to the observed *D*_slow_. The characteristic *Q* dependence of rotational diffusion is visible with no additional contribution at low *Q* but an increase to a plateau if the observation length scale 1/*Q* covers the protein size above *Q* ≈ 0.6 nm^-1^. For larger concentrations in particular ≥ 84 mg/ml the small difference between *D*_slow_(Q) and ${D}_{c}^{s}\left(Q\right)$ (gray area) around *Q* ≈ 0.6 nm^-1^ indicates a full suppression of rotational diffusion. Suppression on the observation time scale means that rotational diffusion might be present, but with very long relaxation times, which cannot make a significant contribution. Rotational correlation times ≈1 µs would contribute less than 2%. This is a direct consequence of fragment collisions that disturb a free rotation of the mAb.

### Slow diffusion: “search” motions and fragment rotation

The additional contribution of a set of similar displacement modes like “search” motions and fragment rotation to the ${D}_{{slow}}(Q)$ using a common amplitude and relaxation time is^64^ (see Methods: mode relaxations)

4 $${D}_{m}\left(Q\right)={a}^{2}/\lambda \frac{{\sum }_{\alpha }{P}_{\alpha }\left(Q\right)}{{Q}^{2}\left[P\left(Q\right)+{a}^{2}{\sum }_{\alpha }{P}_{\alpha }\left(Q\right)\right]}$$

**Fig. 6:**
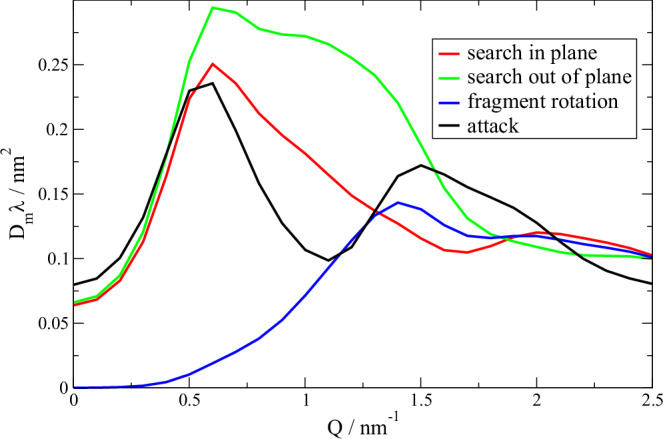
Displacement patterns *D*_*m*_(*Q*)*λ* (dynamic form factors) with rmsd = 0.5 nm per mode resulting from mode displacements of fragments according to Eq. 4 summed over the fragments. Displacements are shown in Fig. 1 with corresponding colors. “Search” motions are the bending motions in plane (red) and out of plane (green). “Attack” motions are the stretching motions (black). Fragment rotation (blue) is an axial rotation of the fragments around the connection line of the CPPC center and their center of mass. Search out of plane and rotational diffusion of the entire mAb (see SI Figure S3) are not well distinguished. The contribution from “attack” motions is visible in Fig. 5 as difference between *D*_slow_ and *D*_cum_ while the summed contribution from “search” motions and fragment rotation are the difference between ${D}_{t}+{D}_{r}$ and *D*_slow_.

From Eq. 4 it is clear that $a$ and $\lambda$ cannot be determined independently. Different displacements result in characteristic patterns (dynamic form factors) in ${D}_{m}\left(Q\right)\lambda$ as presented in Fig. 6 for the previously described iDOF displacement types. “Search” and “attack” motions present a distinct increase to a peak at 0.6 nm^-1^, which represents the distance between fragments. “Attack” motions show a deep minimum with a second maximum, while the dynamic form factor of the “search” motions presents a gradual reduction above 0.6 nm^-1^. In between the different fragments Fab_l_, Fab_m_ and Fc the “attack” motion dynamic form factors present only small variation compared to larger differences for “search” motions (see SI Fig. S3). Fragment rotation contributes at larger *Q* with a peak around 1.4 nm^-1^ that represents the smaller fragment size. The characteristic differences presented in Fig. 6 allow us to discriminate the contributions as spatial information about the movements is encoded in the different shapes.

We have already used the “attack” pattern above for the fast relaxation. Now we use the sum over all “search” motions as one component and all fragment rotations as a second independent component with the same assumed relaxation time of $\lambda$=200 ns. The total contribution of both components corresponds to the area between ${D}_{t}\left(Q\right)+{D}_{r}(Q)$ and ${D}_{{slow}}\left(Q\right)$ and is indicated, including *D*_*r*_(*Q*) as gray area in Fig. 5. The resulting *rmsd u*_*m*_ of all “search” motions and the fragment rotation *rmsd u*_*r*_ were free fit parameters in the model and are presented in Fig. 5 for each concentration. We observe for smaller concentrations *u*_*m*_ ≈ 1 and 3 nm with *u*_*r*_ of 0.83 and 2.81 nm, respectively, indicating strong “search” motions and fragment rotation. The larger values at 50 mg/ml might result from shorter relaxation times $\lambda$ that arise from additional collisions with other fragments. At higher concentrations smaller values of u_r_ could result from increased friction between fragments if the distance is reduced. A systematic variation of all parameters cannot be expected as around 33 mg/ml we observe the transition from protein interactions to fragment interactions. Furthermore, diffusion contributions, just like amplitudes and - here fixed - relaxation times, are observables that depend on the determining parameters of forces and friction, like the fast “attack” motion. For 25 mg/ml we observe large errors for *f*_*r*_ and *u*_*m*_ and a correlation close to 1. This is a result of the similar shape of ${D}_{m}\left(Q\right)$ for “search” motions and rotational diffusion ${D}_{r}\left(Q\right)$ of the entire mAb. This can be understood as each bending of a domain in plane or out of plane can be interpreted as an infinitesimal contribution to rotational diffusion, which on long-time contributes to rotational diffusion. At larger concentration the “search“ motions like rotational diffusion are suppressed as indicated by the small difference between ${D}_{t}\left(Q\right)$ and ${D}_{{slow}}\left(Q\right)$ around Q ≈ 0.6 nm^-1^ in Fig. 5. Fragment rotations remain visible at larger *Q* as these contribute at larger *Q* (see blue line in Fig. 6). The strong slowing down e.g. due to a strong hydrodynamic interaction might be the reason for the vanishing contributions from “search” motions. The contribution of the fragment rotation is also strongly slowed down compared to the free fragment rotational correlation time of ≈41 ns. While a faster fragment rotation with smaller amplitude is possible as we assume $\lambda$=200 ns it should be well separated from the fast “attack” contribution.

### “Attack” motion in the initial slope

As a last step we examine the contribution from the fast “attack” motion on top of ${D}_{{slow}}(Q)$, which can be directly calculated from the values in Table 1 using Eq. 4 and is presented in Fig. 5 as ${D}_{f}\left(Q\right)={D}_{m,{attack}}(Q)$ (dash-dotted line). We can compare the result to a cumulant analysis yielding the diffusion coefficient ${D}_{{cum}}(Q)$ in the initial slope using t < 20 ns (see Methods), which is shown in Fig. 5 as triangles. We observe a characteristic modulation in this model-free analysis that can be reproduced with the additional fast contribution ${D}_{f}(Q)$. The area between ${D}_{{slow}}(Q)$ and ${D}_{{cum}}(Q)$ or respective models (light green area in Fig. 5) is the direct result of the “attack” movements contributing to ${D}_{f}(Q)$. This is reproduced for the larger concentrations with a small overestimation at lower *Q*. Using *H*_*d*_(*Q*) calculated for the translational diffusion as an estimate for HI between the fragments and other mAb (*D*_*f*_(*Q*)→ *H*_*d*_(*Q*)*D*_*f*_(*Q*) dash-double-dot line) we get an improved description that suggest that at lower *Q* HI slow down the “attack” motions. The extrapolation to DLS *Q* suggests that DLS data might be influenced if the HI do not suppress the fast modes at very low *Q*. Standard DLS instruments are not sensitive to these short times—about 100 nanoseconds—and primary relaxation occurs at about 10 microseconds.

Here we explicitly note that for the concentrations > 50 mg/ml the contribution to effective diffusion on short times *t* < 20 ns is to ≈60% translational diffusion. The remaining part are internal motions from fragment dynamics. The rotational diffusion is suppressed. The major contribution to internal motions is from “attack” motions. The fraction decreases for higher *Q* when the observation length scale *2π*/*Q* decreases from fragment distances around 0.6 nm^-1^ and reaches the fragment size with larger contributions of fragment rotation. Experimental methods accessing only shorter timescales <10 ns as e.g., neutron backscattering^24,37^ need to take these effects into account as internal motions largely contribute. Rotational motions of asymmetric proteins cannot be assumed to be hard sphere like above concentrations when the bounding spheres start to touch. Also, NSE measurements limited to timescales comparable to the attack motions <50 ns observe a contribution of internal motions and not only pure D_c_^65,66^.

### Connecting short-time and long-time diffusion

**Fig. 7:**
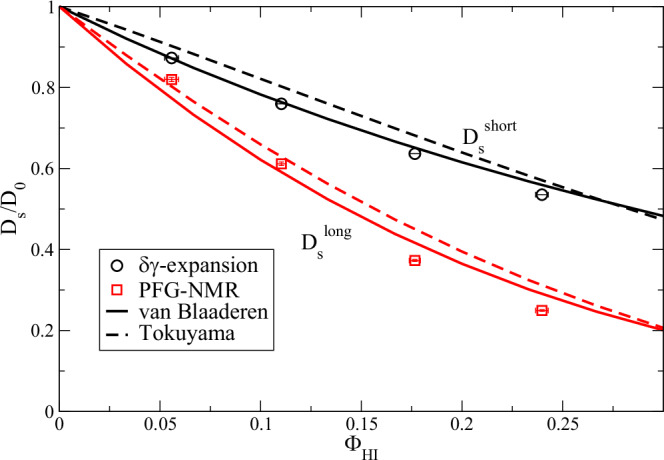
Short and long-time self-diffusion *D*_*s*_/*D*_0_ resulting from the NSE analysis using the δγ-expansion and PFG-NMR for T = 25° C. Errors represent 1-sigma errors from the fit. For comparison we show analytic models from van Blaaderen et al.^67^ and Tokuyama et al.^68^ for short and long-time ${{D}_{s}/D}_{0}$ of interacting hard sphere models. The volume fraction *Φ*_*HI*_ of experimental data refers to the volume fraction used in the δγ-expansion. It should be mentioned that the presented models are not fitted to the data.

Figure 7 presents the evaluated ${D}_{s}^{s}/{D}_{0}$ resulting from the previous analysis and ${D}_{s}^{l}/{D}_{0}$ from PFG-NMR against the volume fraction ${\phi }_{{HI}}$ resulting from the δγ-expansion. The difference between short-time and long-time limits for a particle with radius *a* and diffusion constant ${D}_{0}$ relates to the phenomenon that for Brownian diffusion at short-times the configuration of next neighbors is not changing ($t < {a}^{2}/{D}_{0}$) while for long-times ($t\, \gg \, {a}^{2}/{D}_{0}$) the cage of next neighbors needs to change to allow diffusion for longer distances. The short-time self-diffusion ${D}_{s}^{s}$ can be related to the long-time self-diffusion ${D}_{s}^{l}$ according to van Blaaderen et al.^67^ and Tokuyama et al.^68^ by examining ${D}_{s}/{D}_{0}$ (See SI for details). The theoretical models describe the behavior of hard spheres with direct and hydrodynamic interaction resulting in relations for short and long-time ${D}_{s}/{D}_{0}$. While the work of van Blaaderen et al. is based on the Stokes equation, Tokuyama et al. start from the Navier-Stokes equation. For ${D}_{s}^{s}/{D}_{0}$ we observe an agreement with van Blaaderen et al., which might be related to the similarity to the δγ-expansion and using ${\phi }_{{HI}}$ as X-axis. Nevertheless, also the correspondence to Tokuyama et al. is good. Preconceiving the strong asymmetric shape of the mAb with important internal dynamics the matching of ${D}_{s}^{l}/{D}_{0}$ with theory is good and still within the typical range of other comparisons of experiment and theory^67,68^.

## Discussion

The dynamics of mAb are quite complex with contributions of translational/rotational diffusion and internal fragment dynamics. Looking at the mAb as a colloidal particle, it is surprising how effective the colloidal picture is for the description of translational diffusion if HI is considered by an effective HI interaction radius *R*_*HI*_. We can accurately describe the collective effects at low *Q* combining DLS and NSE. This allows an understanding of the additional internal contributions to the measured effective diffusion and a unique determination of the short-time translational diffusion ${D}_{s}^{s}$. The colloidal picture provides the missing link between short and long-time translational diffusion, which is related to the macroscopic viscosity (${D}_{s}/{D}_{0}\approx \eta /{\eta }_{0}$), a factor that is relevant for the application of mAb as a drug if the corresponding ${\varPhi }_{{HI}}$ is considered. Including the effect of the fast “attack” motions, internal fragment motions can contribute as much as ${D}_{s}^{s}$ to the observed effective diffusion for *t* < 5 ns. The suppression of the overall rotational diffusion is significant and presents a deviation from the colloidal picture for rotational diffusion. A slowing down in the presence of crowding is expected even for hard spheres due to HI^69^. In the case of mAb, the suppression is more noticeable because of the Y-shaped structure and direct interactions with the surrounding fragments, which stop rotation. Consequently, a neighboring fragment cage is constructed, preventing the mAb fragment from escaping due to its linkage to the other fragments. The dynamic crossover from single protein dynamics to linked fragment dynamics around 33 mg/ml coincides with the change in SF when we must switch from protein number density to 3 times larger fragment number density. Both changes indicate a relevant change of interactions because of the remarkable mAb structure.

The fragment dynamics is directly related to the structure of the linker. “Search” motions as well as the rotational diffusion of the entire mAb are suppressed if concentration is increased from dilute to physiologically relevant levels and larger. Remarkably, the “attack” pattern exposing the antigen binding site and the fragment rotation is well preserved within these concentration ranges. Within the picture of caging, this is reasonable as dislocations along the fragment axis and fragment rotations do not need a significant rearrangement of the next neighbor cage. Here, it should be unimportant if the crowding conditions are due to other mAb or due to proteins in the plasma or a virus surface. While the details of the interaction between fragments and crowders will be relevant for HI and the weakening of the entropic spring, the general picture should not depend on the type of crowders.

The “attack” motion itself can support binding of the paratope to the epitope of an antigen. Assuming an activation energy barrier the additional energy stored in the entropic spring can help to overcome the energy barrier or clamp the epitope. Rotational motions of the fragment allow a rearrangement if the orientation is not perfect. “Attack” motions support binding and consequently the strain from the linker also supports release^70^. This mechanism might also be relevant for the Fc fragment to be recognized by Fc receptors. Ig appear in five classes as monomers (IgD, IgE and IgG) and with additional joining chains linking Fc fragments as dimers (IgA) or pentamers (IgM) with a conserved scheme of the Y-shape structure. The described mechanism of fragment mobility with “search” and “attack” motions may be one reason why Ig have a conserved scheme of fragments connected by flexible linkers promoting binding^14^. Detailed knowledge of the underlying molecular mechanisms may allow rational design of the linker region or tuning of the surface properties to improve mAb as therapeutic drugs. Our findings are highly relevant for mAb formulation science and the development of mAb for pharmaceutical administration.

## Materials and Methods

### Small-angle scattering

The scattered intensity ${I\left(Q,c\right)=c/{M}_{w}P\left(Q\right)S}^{{\prime} }\left(Q,c\right)$ of a protein in solutions with concentration *c* and protein molecular weight ${M}_{w}$ can be split into a form factor *P(Q)* and an apparent structure factor (SF) ${S}^{{\prime} }\left(Q\right)$. The lower concentrations can be used to extrapolate the concentration-scaled data to infinite dilution, yielding the form factor *P*$\left(Q\right)=\left\langle \rho \left(Q\right){\rho }^{* }\left(Q\right)\right\rangle$ *as* ${S}^{{\prime} }\left(Q,c\to 0\right)=1$. The scattering amplitude is $\rho \left(Q\right)={\sum }_{k}{b}_{k}{e}^{{iQ}{r}_{k}}$ of protein atoms at position *r*_k_ with scattering length contrast *b*_k_. With known *P(Q)* we can extract the apparent ${S}^{{\prime} }\left(Q\right)$, which is shown in the inset of Fig. 2. The apparent SF is ${S}^{{\prime} }\left(Q,c\right)=1+\beta \left(Q\right)\left(S\left(Q,c\right)-1\right)$ taking the mAb asymmetric shape into account by the asymmetry factor $\beta (Q)={|\langle \rho (Q)\rangle |}^{2}/\langle {|\rho (Q)|}^{2}\rangle$^71^. The SF *S*(*Q, c*) describes the interaction between the mAb. S(Q) is employed as a shorthand for S(Q, c).

### Mode relaxations

In a small displacement approximation of normal mode displacements, we get^39,42,64^.

$$I\left(Q,t\right)/I\left(Q,0\right)=\left(\left(1-A\right)+A\left(Q,t\right)\right){e}^{-{Q}^{2}{D}_{{eff}}\left(Q\right)t}$$

$$A\left(Q,t\right)=\frac{{\sum }_{\alpha }{{e}^{-t/{\lambda }_{\alpha }}a}_{\alpha }^{2}{P}_{\alpha }\left(Q\right)}{P\left(Q\right)+{\sum }_{\alpha }{a}_{\alpha }^{2}{P}_{\alpha }\left(Q\right)}$$

5 $${P}_{\alpha }\left(Q\right)=\left\langle {\sum }_{k.l}{b}_{k}{b}_{l}{e}^{{iQ}\left({r}_{k}-{r}_{l}\right)}\left({{{\boldsymbol{Q}}}}\cdot {{{{\boldsymbol{d}}}}}_{k}^{\alpha }\right)\left({{{\boldsymbol{Q}}}}\cdot {{{{\boldsymbol{d}}}}}_{l}^{\alpha }\right)\right\rangle$$

Here, *Q* is the scattering vector, ${{{{\boldsymbol{d}}}}}_{k}^{\alpha }$ is the atomic displacement of atom *k* in a mode *α* with eigenvalue 1/${\lambda }_{\alpha }$, *r*_k_ is the position and *b*_k_ the scattering contrast of respective atoms. *P*_*α*_(*Q*) is a coherent mode form factor that is characteristic of different eigenmodes. The factor ${a}_{\alpha }$ is a scaling factor for displacements ${{{{\boldsymbol{d}}}}}_{k}^{\alpha }$ and can be chosen equal for a set of similar modes α for fitting. Assuming overdamped relaxation of the modes with common relaxation time *λ* and factor $a$ allows to simplify *A*(*Q*,*t*) of included modes like.

6 $$A\left(Q,t\right)={e}^{-{{{\rm{t}}}}/\lambda }A\left(Q\right)$$

with

7 $$A\left(Q\right)=\frac{{a}^{2}{\sum }_{\alpha }{P}_{\alpha }\left(Q\right)}{P\left(Q\right)+{{{{\rm{a}}}}}^{2}{\sum }_{\alpha }{P}_{\alpha }\left(Q\right)}$$

to allow a fit with two parameters and precalculated mode form factors *P*_α_(*Q*).

The diffusion coefficient $D(Q)$ in the initial slope of Eq. 5 comprising all contributions to diffusion at short times can be calculated from the cumulant^72,73^.

8 $$\tfrac{\partial }{\partial t}{{{\mathrm{ln}}}}\left(I\left(Q,t\right)\right){|}_{t\to 0}={-Q}^{2}D(Q)$$

*D*(*Q*) can be determined experimentally at short times by a cumulant analysis, fitting $I\left(Q,t\right)\approx A\exp (-{Q}^{2}{Dt}+\frac{1}{2}k{t}^{2})$) with an amplitude *A* and *k* describing deviations. From Eq. 8 the additional diffusion from a set of modes contributing to Eq. 6 yields ${D}_{m}\left(Q\right)=A(Q)/\lambda {Q}^{2}$ resulting in the additional contribution to diffusion as given in Eq. 4. Root mean square displacements (*rmsd*) are calculated from displacement vectors and factor *a* as ${{{{\boldsymbol{u}}}}}_{\alpha }=a{\sum }_{k}\left|{{{{\boldsymbol{d}}}}}_{k}^{\alpha }\right|/N$ with the number of atoms *N*.

### Ornstein Uhlenbeck process

The coherent intermediate scattering function for the internal motions ${I}_{{{\mathrm{int}}}}\left(Q,t\right)$ along normal modes in the high friction limit is^40,74,75^

9 $${I}_{{OU}}\left(Q,t\right)=\left\langle {\sum }_{k,l}{b}_{k}{b}_{l}\exp \left(i{{{\boldsymbol{Q}}}}\left({{{{\boldsymbol{R}}}}}_{k}^{{eq}}{-{{{\boldsymbol{R}}}}}_{l}^{{eq}}\right)\right){f}_{{kl}}\left({{{\boldsymbol{Q}}}},{\infty }\right){f}_{{kl}}^{{\prime} }\left({{{\boldsymbol{Q}}}},{{{\rm{t}}}}\right)\right\rangle$$

The time-independent Debye–Waller like factor is

10 $${f}_{{kl}}\left({{{\boldsymbol{Q}}}},{\infty }\right)=\exp \left(-{\sum }_{j={modes}}\frac{1}{2}\left({\left({{{{\boldsymbol{d}}}}}_{{jk}}{{{\boldsymbol{Q}}}}\right)}^{2}+{\left({{{{\boldsymbol{d}}}}}_{{jl}}{{{\boldsymbol{Q}}}}\right)}^{2}\right)\right)$$

related to displacements ${{{{\boldsymbol{d}}}}}_{{jk}}=\sqrt{{k}_{b}T/{k}_{j}}{\hat{{{{\boldsymbol{e}}}}}}_{{jk}}$ in a harmonic potential with effective force constant ${k}_{j}={m}_{k}{\omega }_{j}^{2}$ for mode j. The time-dependent part is

11 $${f}_{{kl}}^{{\prime} }\left(Q,t\right)=\exp \left({\sum }_{j={modes}}\left({{{{\boldsymbol{v}}}}}_{{jk}}{{{\boldsymbol{Q}}}}\right)\left({{{{\boldsymbol{v}}}}}_{{jl}}{{{\boldsymbol{Q}}}}\right)\exp \left(-{\lambda }_{j}t\right)\right)$$

with displacements ${{{{\boldsymbol{v}}}}}_{{jk}}=\sqrt{{k}_{b}T/\left({\lambda }_{j}{\Gamma }_{j}\right)}{\hat{{{{\boldsymbol{b}}}}}}_{{jk}}$ of relaxation time 1/*λ*_*j*_ and effective friction *Γ*_*j*_. Normal mode analysis of a structural atomic model results in Brownian normal modes with related eigenvalues fixing ${\omega }_{j}^{2}$ and ${\lambda }_{j}$^74,75^. Instead, we use here the previously described motional “attack” pattern and use the additional requirement that the effective force *k*_*j*_ in the effective potential and the force related to friction are equal with ${k}_{j}={\lambda }_{j}{\Gamma }_{j}$.

### δγ-expansion

The hydrodynamic function $H(Q)={H}_{{{{\rm{d}}}}}(Q)+{D}_{s}^{s}/{D}_{0}$ for spherical particles of radius *R* can be approximated by the *δγ*-expansion of Beenakker and Mazur^76,77^ with the distinct contribution.

12 $${H}_{d}\left(Q\right)= 	\frac{3}{2\pi }{\int }_{0}^{{\infty }}d\left({Rk}\right)\frac{{\sin }^{2}\left({R}_{{HI}}k\right)}{{\left({ak}\right)}^{2}\left[1+{\Phi }_{{{{\rm{HI}}}}}{S}_{\gamma }\left({R}_{{HI}}k\right)\right]}\\ 	\,\times {\int }_{-1}^{1}{dx}\left(1-{x}^{2}\right)S\left(\left|{{{\boldsymbol{Q}}}}-{{{\boldsymbol{k}}}}\right|-1\right)$$

and the self-part describing the change in short-time self-diffusion

13 $${D}_{s}^{s}\left(\Phi \right)/{{{{\rm{D}}}}}_{0}=\frac{2}{\pi }{\int }_{0}^{{\infty }}{dtsin}{c}\,^{2}\left(t\right){\left[1+{S}_{\gamma }\left(t\right)\right]}^{-1}$$

$x$ is the angle between wave vectors ***Q*** and ***k***, while ${S}_{\gamma }$ is a known function given in ref. ^78^ Particle correlation and the associated particle interactions enter the distinct part *H*_*d*_ through *S*(*Q*) measured by SAXS. The hydrodynamic interaction (HI) enters as the mobility of a sphere with the geometrical radius ${R}_{{HI}}$. The corresponding HI volume fraction is ${\Phi }_{{{{\rm{HI}}}}}=n4\pi {R}_{{HI}}^{3}/3$ with particle number density *n*. The shape of *H*_d_(*Q*) can be estimated as *H*_d_(*Q*) ≈ 1 + (*S*(*Q*)-1)*f with *f* < 1, compensating partly the modulation of 1/*S(Q)* in the *H*(*Q*)/*S*(*Q*) correction. We fit here the radius *R*_*HI*_ = *R*_h_·*f*_c_ as a fraction *f*_c_ of the hydrodynamic radius *R*_h_ of the diluted mAb.

### Samples

NISTmAb Primary Sample (PS 8670) was obtained from NIST (Gaithersburg, MD, US). NISTmAb was received at 100 mg/ml as frozen solution, thawed and gently shaken. mAb solutions were diluted by 1:10 with the final D_2_O buffer and up-concentrated in a Vivaspin 20 concentrator (Sartorius, Goettingen) with a 10 kDa MW cutoff. The dilution-up-concentrating was six times repeated to remove impurities from the initial buffer and H_2_O. The buffer solution contained 15 mM acetic acid and 40 mM NaCl at pH 5 resulting in an ionic strength of 50 mM. The buffer was chosen within the project after systematically testing protein solutions under varying temperature, pH and ionic strength without additional excipients to find stable, aggregation-free conditions. FPLC showed no significant appearance of dimers (<1%). DLS and PFG-NMR verified reproducibility of measurements over several days including temperature changes. *pH* was adjusted using DCl to *pH* meter reading of 5. Final concentrations were adjusted by up concentration or dilution to the desired concentration. Concentration was determined by a Nanodrop 2000c UV spectrometer (Thermo Fischer, Darmstadt) with an extinction coefficient of 1.42 ml/mg·cm^79^. Temperature of all measurements was 25 °C if not explicitly indicated differently. NISTmAb reference material was developed as an industry standard with high stability and homogeneity that present a low amount of aggregation. Detailed analysis regarding NISTmAb can be found in Yandrofski et al.^53^.

#### Neutron Spin Echo

NSE measurements were performed at the instrument IN15^80,81^ (ILL, Grenoble). Measurements were conducted at wavelength $\lambda$ of 1.2 and 0.8 nm resulting in a maximum time ${t}_{\max } \sim {\lambda }^{3}$ of 99–335 ns, respectively. Buffer was measured as background with similar measurement time as the samples and subtracted by usage of the instrument software.

#### Small angle X-ray scattering (SAXS)

experiments were performed on the in-house instrument SAXSpace (Anton-Paar, Austria, Cu-Kα, λ = 0.154 nm) equipped with a Kratky block-collimation system and a CCD camera. The beam slit length was 20 mm. mAb solutions from NSE preparation, respectively, diluted samples for lower concentrations and background buffer were filled consecutively into the same sealed quartz capillary (1 mm diameter) and measured for 1 h (720 times 5 s frames). Additionally empty cell, empty beam and a dark image were measured. Data frames were filtered for cosmic rays and dead pixel. Averaged data were corrected for transmission and dark counts, empty cell and buffer scattering were subtracted in analogy to Brûlet et al.^82^. Finally, corrected measurements were desmeared by using the Lake algorithm as improved by Vad and Sager^83^. To achieve better statistics at larger Q, a Q binning was applied with equal distance of points on a log scale. All steps are implemented in Jscatter^84^.

### Pulsed-Field Gradient NMR (PFG-NMR)

The PFG NMR measurements were performed using a Varian 600 MHz system equipped with a diffusion ^1^H probe head. The attenuation of the spin echo signal from a pulse sequence containing a magnetic field gradient pulse is used to measure the large-scale translational diffusion ${D}_{s}^{l}$ of the molecules (hydrogens) in the sample on time scales from ten to a few hundred milliseconds. During this period, the diffusion of hydrogen occurs over distances that are approximately hundreds of nanometers. Diffusion spin echo decays were measured using a standard stimulated echo (STE) pulsed field gradient sequence^85^ with convection compensation in the temperature range from 15 °C to 35 °C. The diffusion time ∆ was equal to 20 ms. The gradient pulse length δ was 2 ms. The integrated spin echo decay was determined as a function of magnetic field gradient. Errors are determined as 1-sigma error from the fit (see SI).

### Dynamic light scattering

DLS was measured using a Zetasizer Nano ZS (Malvern) at a wavelength of 633 nm with a scattering angle of 173° (Q = 0.026 nm^-1^) and analysed using the built in NNLS algorithm. Reported DLS values are determined as mean of at least ten measurements on the same sample to check stability.

### Statistics and Reproducibility

The fittings of the described models were performed with nonlinear least-square methods minimizing reduced weighted χ^2^. Corresponding errors were defined as the resulting 1-sigma errors.

### Data analysis

Data analysis was done using the Python-based open-source project Jscatter version 1.72^84^ using a new module *bio*. *bio* allows protein and DNA modeling of SAXS/SANS measurements and respective dynamics based on atomic structures.

### Supporting Information

SAXS form factors, Structure factors, NSE spectra, *D*_*m*_(*Q*), short and long-time ${{D}_{s}/D}_{0}$, PFG-NMR, filename: mAb_AttackandSearch.pdf.

### Reporting summary

Further information on research design is available in the Nature Portfolio Reporting Summary linked to this article.

## Supplementary information

Supplementary Material

Reporting Summary

## Data Availability

The authors declare that the data supporting the findings of this study are available within the paper, its supplementary information files. Raw data for figures are available as download^86^.

## Code availability

All code is available in GitLab at the following link: https://gitlab.com/biehl/jscatter with the used version 1.7.2 deposited at 10.5281/zenodo.4662911^87^.
