# Supplementary material for: Diffusion, search and attack motions of antibodies
Source: Commun Biol. 2025 Oct 13;8:1461. doi: 10.1038/s42003-025-08995-9 (PMC12518842; doi:10.1038/s42003-025-08995-9)
Supplement: Supplementary file 1 — Supplementary Material [file 42003_2025_8995_MOESM1_ESM.pdf]

# Diffusion, search and attack motions of antibodies

Ralf Biehl,[1*] Margarita Kruteva,[1] Orsolya Czakkel[2], Ingo Hoffmann,[2] Dieter Richter[1], Andreas M. Stadler[1,3,4]

[1]Jülich Centre for Neutron Science JCNS, Forschungszentrum Jülich GmbH, 52425 Jülich, Germany

[2]Institut Max von Laue-Paul Langevin (ILL), 71 Avenue des Martyrs, CS 20156, F-38042 Grenoble Cedex 9, France

[3]Institute of Physical Chemistry, RWTH Aachen University, Landoltweg 2, 52056 Aachen, Germany

[4]LINXS Institute of advanced Neutron and X-ray Science (LINXS), IDEON Building: Delta 5, Scheelevägen 19, 223 70 Lund, Sweden

## Supporting information

### *SAXS Formfactors*

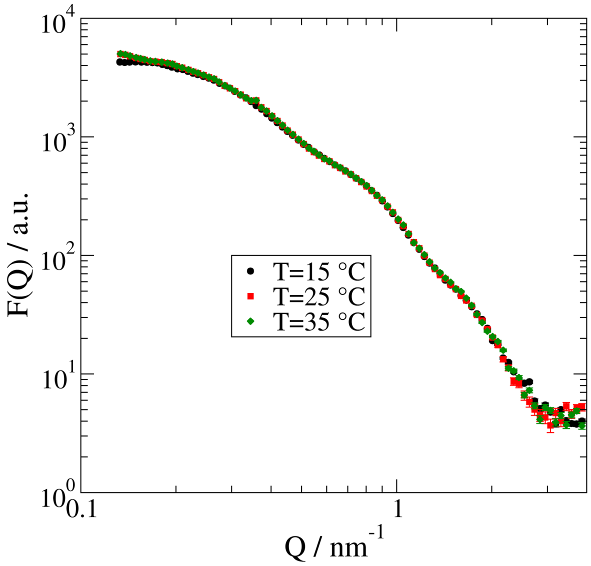


*Figure S1: SAXS formfactor extracted from concentration series measured at different temperature. Error bars represent standard deviation of the mean.*

## *Structure factors*

### Rescaled mean spherical approximation (RMSA)

Structure factor for a screened coulomb interaction (single Yukawa) in rescaled mean spherical approximation (RMSA) according to Hayter-Penfold[1,2]. Consider a scattering system consisting of macro ions with radius R and counter ions of density $n_i$ and charge $z_i$ in a solvent. The potential is:

$$V(r)/kT = \begin{bmatrix} \infty & r \leq 1 \\ k_1 \left[\frac{e^{-\sigma x}}{x}\right] & r > 1 \end{bmatrix}$$

$x = r/R$ dimensionless parameters of distance r, rescaled inverse screening length $\sigma = sR$ with screening length $s$ and $s^2 = 4\pi\lambda_B \sum_i n_i z_i$. $\lambda_B = 0.71$ nm is the Bjerrum length. $k_1$ is the contact potential in kT units.

### Two Yukawa structure factor (2Y)

The double Yukawa potential of particles with radius R at distance r' is

$$V(r)/kT = \begin{bmatrix} \infty & r \leq 1 \\ -k_1 \left[\frac{e^{-z_1(r-1)}}{r}\right] - k_2\left[\frac{e^{-z_2(r-1)}}{r}\right] & r > 1 \end{bmatrix}$$

with $z_i=1/s_I$ as inverse screening length ($s_I$ as screening length), reduced distance r=r'/R and potential $k_i$ at the surface. For $k_i>0$ we have attraction while $k_i<0$ means repulsion.

Liu et al. describe the corresponding structure factor S(Q) within the MSA closure.[3]

### Structure factor fit parameters

The SAXS structure factor is fit by the RMSA SF for conc. <=25 mg/ml and by the two Yukawa (2Y) SF for higher concentrations. RMSA is fit with common parameters for all concentrations while 2Y is fit with concentration dependent potentials $k_1$ and $k_2$. The large errors for RMSA $k_1$ at a low value indicates that the potential is close to a hard sphere potential with $k_1=0$ and the repulsive interaction has minor contributions. Nevertheless, a hard sphere potential (Percus-Yevick (PY) potential[4,5]) does not fit with same quality and the RMSA correction improves the fit. The parameters for 2Y fits have also larger errors that indicate weaker repulsive and attractive interactions, here indicated more by the short screening length. The main change is the increased concentration (x3). Again, a good fit is only reached by using a 2Y potential instead of a PY or RMSA indicating significance for 2Y. The missing strong characteristics, like a low Q cluster peak for the 2Y potential[3], create parameter dependencies which lead to the larger errors.

*Supplementary table 1 Fit parameters from structure factor fits of SAXS data shown in the main article. Shaded boxes indicate simultaneous fit with a single parameter where only concentration varies. Errors represent 1-sigma errors from the fit.*

| conc. [mg/ml] | R [nm] | $k_1$ [kT] | $s_1$ [nm] | $k_2$ [kT] | $s_2$ [nm] | SF |
|---|---|---|---|---|---|---|
| **5** | 4.6±0.07 | 0.65±0.7 | 6.5±3.6 | - | | RMSA |
| **10** | | | | - | | |
| **15** | | | | - | | |
| **25** | | | | - | | |
| **50** (x3) | 1.59±1.2 | 15.4±30 | 0.37±0.02 | -8±3 | 0.76±0.8 | two Yukawa |
| **84** (x3) | | 14.1±27 | | -9±4 | | |
| **154** (x3) | | 9±17 | | -6±3 | | |

# *NSE spectra*

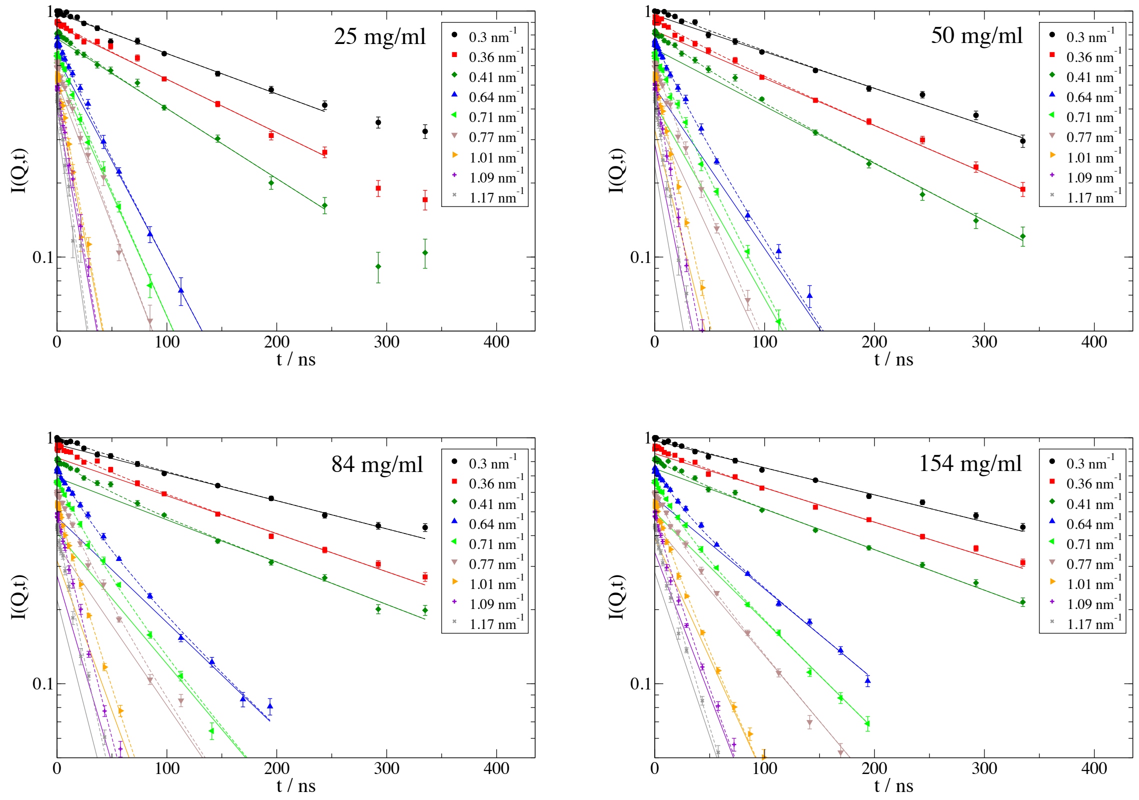


*Figure S2: NSE data with corresponding fits. The data are shifted consecutively for by a factor 0.9 for a clearer picture. Broken lines show the fit result as described in the main text. Solid lines describe the long-time diffusion (slow relaxation) extrapolated to short times to highlight the non-single exponential character of the measured data. Error bars represent standard deviation of the mean.*

## $D_m(Q)$ for specific fragments

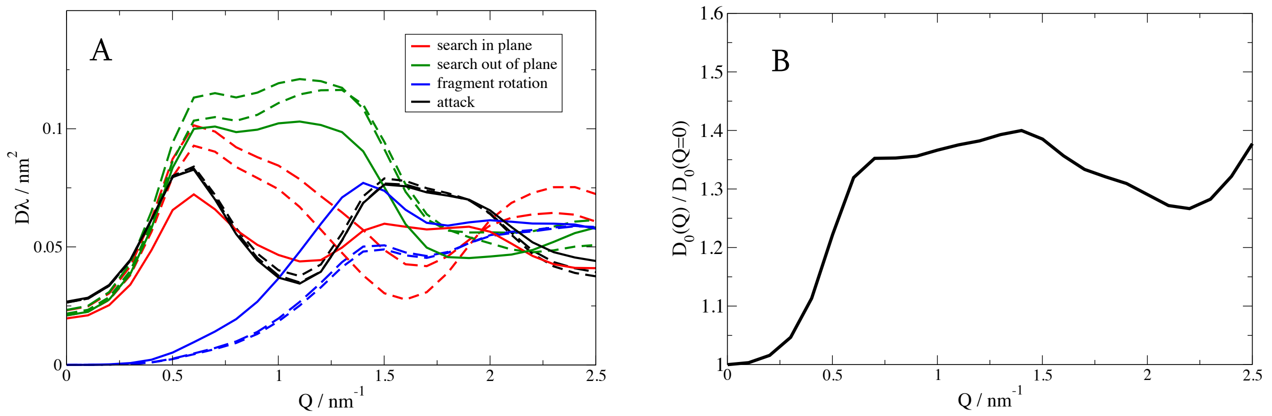


*Figure S3 A: Displacement patterns $D_m(Q)\lambda$ with rmsd 0.5 nm per mode resulting from mode displacements of Fc fragment (solid lines) and Fab fragments (dashed lines: Fab$_l$ short; Fab$_m$ long) according to equ. 3. "Search" motions are the bending motions n plane (green) and out of plane (red). "attack" motions are the stretching motions (light green). Fragment rotation is an axial rotation of the fragments around the connection line of the CPPC centre and their centre of mass. Search out of plane and rotational motions are not well distinguished. B: $D_0(Q)/D_0(Q=0)$ presenting the increased diffusion due to rotational diffusion in the dilute case.*

## Short and long-time self-diffusion

van Blaaderen[6] with volume fraction Φ

$$D_s^s = D_0 \frac{1-\Phi}{1+3/2\Phi}$$

$$D_s^l = D_0 \frac{(1-\Phi)^3}{1+3/2\Phi+2\Phi^2+3\Phi^3}$$

Tokuyama & Oppenheim[7]

$$\mathrm{D_s^s} = \mathrm{D_0}/\big(1+\mathrm{h}(\Phi)\big)$$

$$h(\Phi) = \frac{2\mathrm{b}^2}{(1-\mathrm{b})} - \frac{\mathrm{c}}{(1+2\mathrm{c})} - \frac{\mathrm{bc}(2+\mathrm{c})}{(1+\mathrm{c})/(1-\mathrm{b}+\mathrm{c})}$$

with $b = (9\Phi/8)^{0.5}$ and $c = 11\Phi/16$

$$D_s^l = \frac{D_0(1-9/32\Phi)}{(1+h+\Phi/\Phi_0/(1-\Phi/\Phi_0)^2)}$$

With $\Phi_0 = (4/3)^3/(7ln(3)-8ln(2)+2)$

## *PFG NMR*

The high-resolution NMR spectra of the mAb in acetate buffer at 25ºC demonstrate presence of exchanged water hydrogen (HDO), mAb signal and acetate buffer hydrogens (see exemplary spectra on the Fig.S3). The area indicated by the rose rectangle was used for integration to the avoid the residual water hydrogen contribution.

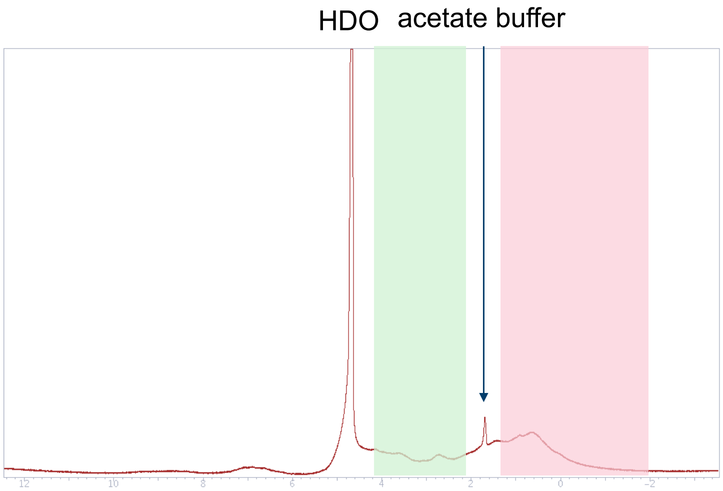


*Fig.S4 High resolution NMR spectra of the mAb in acetate buffer at 25ºC. The areas of the mAb NMR signal are shown by colored areas.*

Integrated amplitudes vs. magnetic field gradient are presented in the Fig.S5. Assuming Fickian diffusion and the validity of the Stoke-Einstein relation a linear relation between mean square displacements and time the amplitude decays were described by equation

$$I(q^2)/I(0) \approx \exp\left(-q^2(\Delta - \delta/3)D_s^l\right)$$

Here the wave number $q$ is defined as $q = \gamma\delta g$ with $\gamma$ as the gyromagnetic ratio for hydrogens, $\Delta=$ 20 ms is the diffusion time and the magnetic field gradient g. The gradient pulse length $\delta$ was set to 2 ms.

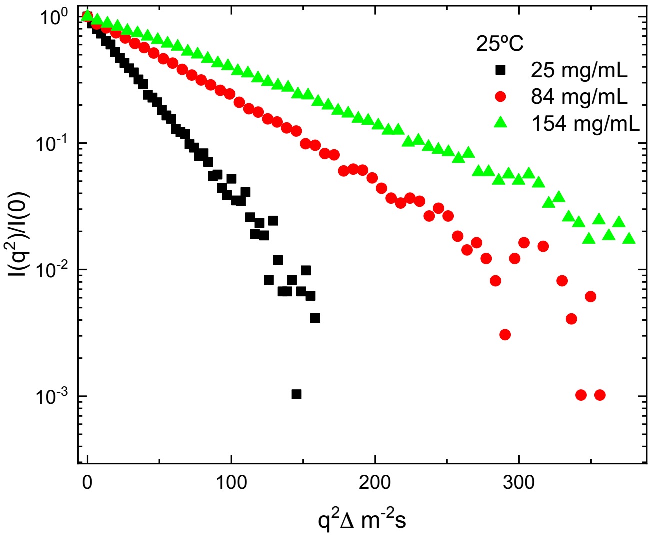


*Fig.S5 NMR signal amplitude decay vs. wave number measured for 3 concentrations at 25ºC.*

## *References*

(1) Hansen, J.-P.; Hayter, J. B. A Rescaled MSA Structure Factor for Dilute Charged Colloidal Dispersions. *Mol Phys* **1982**, *46* (3), 651–656. https://doi.org/10.1080/00268978200101471.

(2) Hayter, J. B.; Penfold, J. An Analytic Structure Factor for Macroion Solutions. *Mol Phys* **1981**, *42* (1), 109–118. https://doi.org/10.1080/00268978100100091.

(3) Liu, Y.; Chen, W. R.; Chen, S. H. Cluster Formation in Two-Yukawa Fluids. *Journal of Chemical Physics* **2005**, *122* (4), 044507. https://doi.org/10.1063/1.1830433.

(4) Wertheim, M. S. Exact Solution of the Percus-Yevick Integral Equation for Hard Spheres. *Phys Rev Lett* **1963**, *10* (8), 321–323. https://doi.org/10.1103/PhysRevLett.10.321.

(5) Percus, J. K.; Yevick, G. J. Analysis of Classical Statistical Mechanics by Means of Collectice Coordinates. *Phys. Rev.* **1958**.

(6) Van Blaaderen, A.; Peetermans, J.; Maret, G.; Dhont, J. K. G. Long-time Self-diffusion of Spherical Colloidal Particles Measured with Fluorescence Recovery after Photobleaching. *J Chem Phys* **1992**, *96* (6), 4591–4603. https://doi.org/10.1063/1.462795.

(7) Tokuyama, M.; Oppenheim, I. Dynamics of Hard-Sphere Suspensions. *Phys Rev E* **1994**, *50* (1), R16. https://doi.org/10.1103/PhysRevE.50.R16.
